# Community structure and insecticide resistance of malaria vectors in northern-central Myanmar

**DOI:** 10.1186/s13071-022-05262-2

**Published:** 2022-05-03

**Authors:** Daibin Zhong, Pyae Linn Aung, Maung Maung Mya, Xiaoming Wang, Qian Qin, Myat Thu Soe, Guofa Zhou, Myat Phone Kyaw, Jetsumon Sattabongkot, Liwang Cui, Guiyun Yan

**Affiliations:** 1 Program in Public Health, College of Health Sciences, University of California at Irvine, Irvine, CA 92697 USA; 2 Myanmar Health Network Organization, Yangon, Myanmar; 3 Medical College, Lishui University, Zhejiang, China; 4 Mahidol Vivax Research Unit, Faculty of Tropical Medicine, Mahidol University, Bangkok, Thailand; 5 Department of Internal Medicine, Morsani College of Medicine, University of South Florida, Tampa, FL 33612 USA

**Keywords:** *Anopheles* malaria vectors, Community structure, Ribosomal internal transcribed spacer 2, Insecticide resistance, *Kdr* mutation, Myanmar

## Abstract

**Background:**

Myanmar is one of the six countries in the Greater Mekong Subregion (GMS) of Southeast Asia. Malaria vectors comprise many *Anopheles* species, which vary in abundance and importance in malaria transmission among different geographical locations in the GMS. Information about the species composition, abundance, and insecticide resistance status of vectorial systems in Myanmar is scarce, hindering our efforts to effectively control malaria vectors in this region.

**Methods:**

During October and November 2019, larvae and adult females of *Anopheles* mosquitoes were collected in three sentinel villages of Banmauk township in northern Myanmar. Adult female mosquitoes collected by cow-baited tent collection (CBTC) and adults reared from field-collected larvae (RFCL) were used to determine mortality rates and knockdown resistance (*kdr*) against deltamethrin using the standard WHO susceptibility test. Molecular species identification was performed by multiplex PCR and ITS2 PCR, followed by DNA sequencing. The *kdr* mutation at position 1014 of the voltage-gated sodium channel gene was genotyped by DNA sequencing for all *Anopheles* species tested.

**Results:**

A total of 1596 *Anopheles* mosquitoes from seven morphologically identified species groups were bioassayed. Confirmed resistance to deltamethrin was detected in the populations of *An. barbirostris* (s.l.), *An. hyrcanus* (s.l.), and *An. vagus*, while possible resistance was detected in *An. annularis* (s.l.), *An. minimus*, and *An. tessellatus*. *Anopheles kochi* was found susceptible to deltamethrin. Compared to adults collected by CBTC, female adults from RFCL had significantly lower mortality rates in the four species complexes. A total of 1638 individuals from 22 *Anopheles* species were molecularly identified, with the four most common species being *An. dissidens* (20.5%) of the *Barbirostris* group, *An. peditaeniatus* (19.4%) of the *Hyrcanus* group, *An. aconitus* (13.4%) of the *Funestus* group, and *An. nivipes* (11.5%) of the *Annularis* group. The *kdr* mutation L1014F was only detected in the homozygous state in two *An. subpictus* (s.l.) specimens and in a heterozygous state in one *An. culicifacies* (s.l.) specimen.

**Conclusions:**

This study provides updated information about malaria vector species composition and insecticide resistance status in northern Myanmar. The confirmed deltamethrin resistance in multiple species groups constitutes a significant threat to malaria vector control. The lack or low frequency of target-site resistance mutations suggests that other mechanisms are involved in resistance. Continual monitoring of the insecticide resistance of malaria vectors is required for effective vector control and insecticide resistance management.

**Graphical Abstract:**

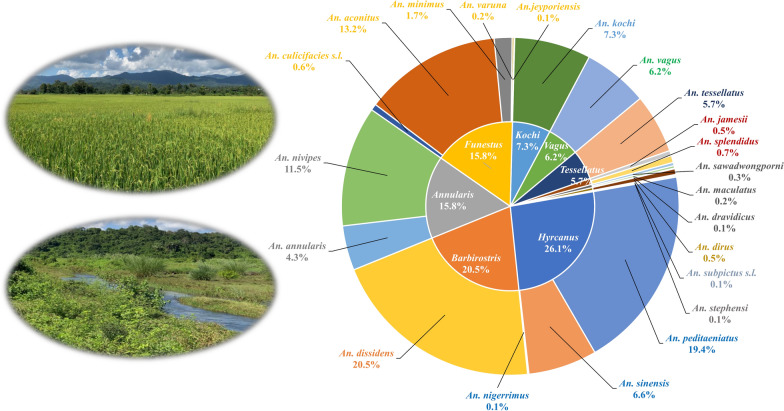

**Supplementary Information:**

The online version contains supplementary material available at 10.1186/s13071-022-05262-2.

## Background

Despite remarkable achievements in the battle against vector-borne diseases over the last decade, malaria remains a major public health problem in many developing countries, especially as progress has been stalled in recent years because of the COVID-19 pandemic. In 2020, there were an estimated 241 million malaria cases and 627,000 deaths from malaria worldwide [1]. Among the six Greater Mekong Subregion (GMS) countries of the WHO Southeast Asia Region, Myanmar had the highest malaria incidence, accounting for 71% of the indigenous cases of malaria in the GMS in 2020 [1].

Malaria control relies heavily on the use of indoor residual spraying (IRS) and long-lasting insecticide-treated nets (LLINs) to prevent mosquitoes from transmitting the malaria parasite to humans [2]. However, the extensive use of chemical insecticides to control disease vectors and other insect pests has resulted in insecticide resistance in many malaria vectors worldwide [3]. The widespread resistance to insecticides has become a significant threat to malaria vector control [4]. In Africa, insecticide resistance and its underlying mechanisms have been well documented in the primary malaria vector *Anopheles gambiae* complex. Resistance to pyrethroids was the most commonly reported, with two main types of known resistance mechanisms: changes at the target site of the insecticide to reduce sensitivity (e.g. knockdown resistance, *kdr*) and increases in the rate of insecticide metabolism due to increased activity or level of detoxification enzyme(s) [5]. In Southeast Asia, however, much less information is available about the status and mechanisms of insecticide resistance in the complex vector systems.

Before the 1980s, regular resistance monitoring of malaria vectors was rarely conducted in the GMS countries. In Vietnam, resistance monitoring indicated that DDT resistance had already existed before 1989 in the malaria vector *An. epiroticus* of the *Sundaicus* Complex. In the 1990s, resistance to pyrethroid insecticides was almost absent in all tested species except in some populations of *An. vagus* and the *Minimus* Complex [6]. There was no evidence of insecticide resistance in malaria vectors in Thailand before 1985. However, from 1986 to 2010, DDT resistance was found in the three primary malaria vectors, *An. dirus* (s.l.), *An. minimus* (s.l.), and *An. maculatus* (s.l.), as well as the three secondary vectors, *An. aconitus, An. philippinensis*, and *An. nivipes*. Permethrin resistance was also detected in a population of *An. minimus* (s.l.) from northern Thailand [6–8]. During 2003 and 2005, in the Mekong Delta region of southern Vietnam, *An. epiroticus* was found to be highly resistant to all pyrethroid insecticides tested. *Anopheles vagus* was found resistant to DDT and several pyrethroids in Vietnam and Cambodia. *Anopheles minimus* (s.l.) showed susceptibility to pyrethroid insecticides in Cambodia, Laos, and Thailand [6].

Recently, *An. hyrcanus* (s.l.) was found highly resistant to multiple insecticides tested (deltamethrin 0.05%, permethrin 0.75% and DDT 4%) in northeastern Thailand, with mosquito mortality ranging from 45 to 87% [9, 10], and at the Myanmar-Thailand border, with mortality ranging from 33 to 57% [11]. Resistance or possible resistance to multiple insecticides was also detected in *An. barbirostris* (s.l.), *An. maculatus*, and *An. vagus* in the Myanmar-Thailand border region, whereas the primary vectors *An. dirus* (s.l.) and *An. minimus* (s.l.) remained susceptible or had suspected resistance to pyrethroids and DDT [9, 11]. In China, high-level resistance to multiple insecticides has been found in *An. sinensis* [12–15].

The mechanisms of resistance to pyrethroid insecticides have been well documented in *An. sinensis* populations from China. Three non-synonymous *kdr* mutations (L1014F, L1014C, and L1014S) have been detected at codon L1014 of the para-type sodium channel gene in *An. sinensis*, and these *kdr* mutant alleles exhibited a patchy distribution in frequency from southern to central China. Near fixation of the *kdr* mutation was detected in populations from central China, but no *kdr* mutation was found in Yunnan Province of southwestern China, suggesting that *kdr* alone is insufficient to predict pyrethroid resistance [16]. Classification and regression tree statistical analysis found that metabolic detoxification was the most important resistance mechanism, whereas target site insensitivity of L1014 *kdr* mutation played a minor role [12]. Whole-genome sequencing (WGS) and RNA-seq analysis identified some transcripts and SNPs associated with phenotypic resistance to deltamethrin [17, 18]. In Thailand and Myanmar, no *kdr* mutation was detected in any *Anopheles* species tested except *An. peditaeniatus* of the *Hyrcanus* group, where the *kdr* mutation L1014S was detected at a low frequency [9, 11]. In Thailand, pre-exposure of *An. hyrcanus* (s.l.) to 4% piperonyl butoxide caused a significant increase in mortality or restored susceptibility, indicating a potential role of oxidases as a detoxifying enzyme resistance mechanism [9, 10].

Malaria vectors are complex in the GMS countries; many *Anopheles* species exist with varying abundance and importance in malaria transmission among different geographical regions. In south-central Vietnam, 24 *Anopheles* species were identified, with the predominant species being *An. dirus* in Binh Phuoc Province and *An. maculatus* in Dak Nong Province [19]. In Ubon Ratchathani Province of northeastern Thailand, 18 *Anopheles* species were identified, with *An. peditaeniatus* as the most abundant species (> 90%) [10]. Rattanarithikul et al. (2006) listed 73 species of *Anopheles* across Thailand [20]. In Myanmar, vector surveillance conducted during the 1970s identified 36 *Anopheles* species, and 10 species were infected with the malaria parasites [21]. However, little information is available about the current community structure and insecticide resistance of the vectorial systems in Myanmar. Such information is needed to guide the vector control practice in this region.

The objectives of this study are to determine *Anopheles* species composition and insecticide resistance status as well as the underlying resistance mechanisms in northern-central Myanmar, to determine the differences between sampling methods (field adult and larval collections) for resistance surveillance, to test the universal *kdr* primer set in different *Anopheles* species collected in Myanmar, and finally to determine the evolutionary relationships among species and species complexes.

## Methods

### Study sites and mosquito collection

**Fig. 1:**
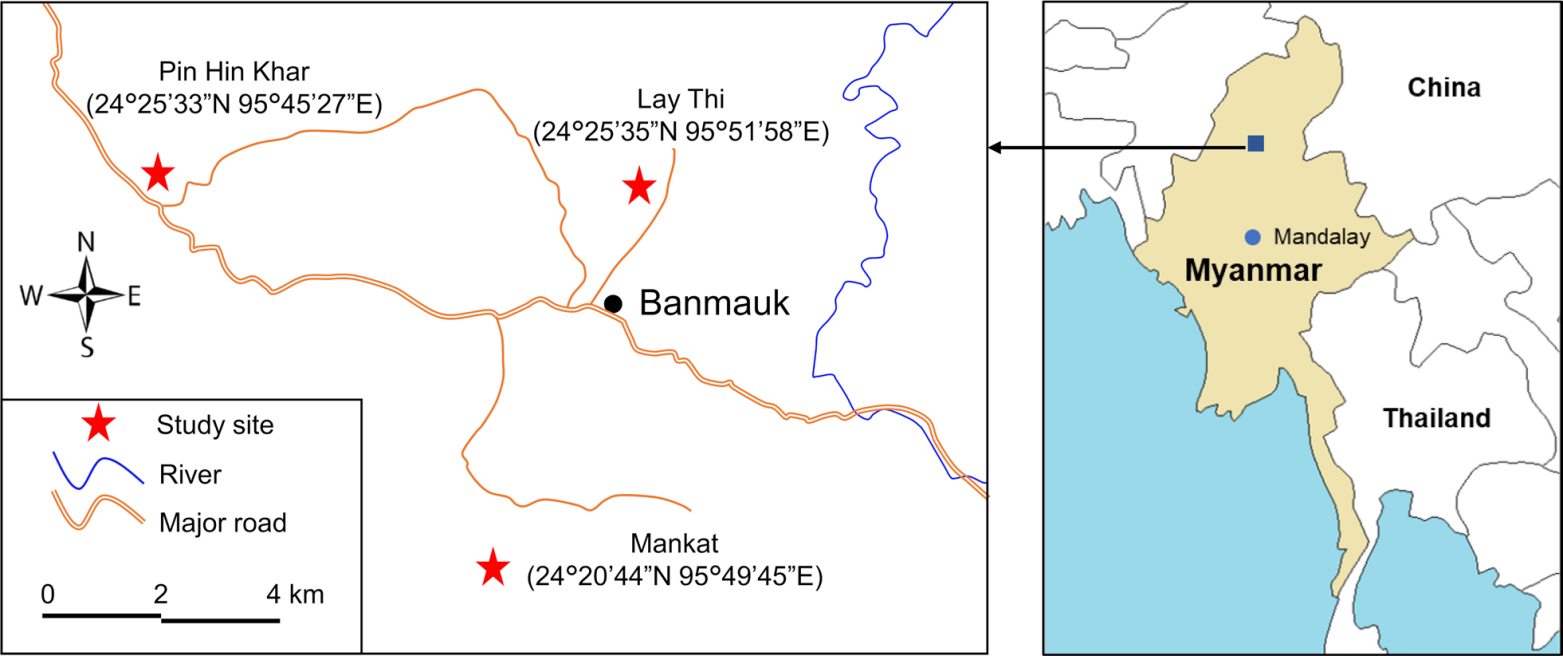
Map of study sites in Banmauk Township, Katha District, Sagaing Region of northern-central Myanmar. Red triangles indicate the locations of the three villages. The map was generated using ArcGIS Pro software based on map source at: www.esri.com

During October–November 2019, entomological surveillance was carried out in three villages located in Banmauk Township, Katha District, Sagaing Region, northern-central Myanmar, namely Pin Hin Khar (24°25′33″N, 95°45′27″ E), Mankat (24°20′44″N, 9549′45″E), and Lay Thi (24°25′35″N, 95°51′58″E) (Fig. 1). In Myanmar, there are three weather seasons: cool dry season (November- February), hot dry season (March to May), and wet (monsoon) season (June to October) [22]. The late rainy season was selected for sampling because of higher mosquito density [23, 24], greater species richness, and relatively little variation among species in their abundance [25]. *Anopheles* adult female mosquitoes were collected every day for 10 days from each village using the cow-baited tent collection (CBTC) method [21, 26]. The collected female mosquitoes belonging to different species were separated by morphology [27] into different cages and reared for 2–3 days to conduct the insecticide susceptibility bioassays. *Anopheles* larvae were collected from different habitat types, including rice fields, rivers, streams, rainwater pools, and other water habitats. To minimize the sampling of siblings, no more than ten specimens (larvae and pupae) were collected from each habitat using a standard 350-ml capacity mosquito dipper. All the randomly selected larval habitats were at least 50 m away from each other. All the collected larvae and pupae were reared to adults (referred to as RFCL) under standard insectary conditions at 27 ± 1 °C, approximately 80% relative humidity, and a photoperiod of 12:12 (L:D) h. The emerged adult females were provided with cotton balls soaked in an 8% glucose solution. Non-blood-fed females 3–5 days post-emergence were separated by morphology into different cages and used for the bioassay as described below. Adult female *Anopheles* were individually transferred to a clean and transparent plastic tube using a HEPA Filter Mouth Aspirator (John W. Hock Company, Gainesville, FL, USA). The morphological identification of *Anopheles* individuals was performed while the mosquito was alive (a brief chill on ice if needed) following the taxonomic keys [27].

### Insecticide susceptibility bioassay

The adult female *Anopheles* mosquitoes obtained from both the CBTC and RFCL collections were used to conduct bioassays following the standard WHO insecticide susceptibility tube-test procedures for malaria vectors [5] to determine their susceptibility to the pyrethroid insecticide deltamethrin (0.05%). Test paper treated with silicone oil was used as the control. For each morphologically identified *Anopheles* species group, female mosquitoes with sample size > 50 were tested with 20–25 mosquitoes per tube and two control tubes without insecticide. The number of knocked-down mosquitoes was recorded at 0, 10, 15, 20, 30, 40, 50, and 60 min after mosquitoes were transferred into test tubes. After 1 h of exposure to the insecticide, the females were transferred into holding tubes, provided with cotton balls soaked in 8% glucose, and kept at 28 °C with a relative humidity of 80%. Mortality was recorded after a 24-h recovery period. Tests were replicated at least twice if enough specimens were collected. The bioassay results were interpreted per WHO guidelines updated in June 2018: confirmed resistance (mortality < 90%), possible resistance (mortality between 90 and 98%), and susceptible (mortality ≥ 98%) [5]. After bioassays, all specimens were individually transferred into a 1.5-ml Eppendorf tube containing silica gel and cotton wool and preserved at − 20 °C for further molecular species identification.

### DNA extraction

All the bioassayed specimens (*n* = 1596) and untested samples (*n* = 42; all species with samples insufficient for the replicate test) were used for DNA extraction and molecular identification of *Anopheles* species. DNA extraction was performed by a rapid and simple method following the published protocol with modifications [28]. Briefly, a single leg of an ethanol-washed mosquito was transferred into a 1.5-ml Eppendorf tube containing 475 µl l × PBS solution and 25 µl 10% saponin solution. The sample was mixed well by gentle vortexing and centrifuged at 18,000 g for 10 min. After discarding the liquid with a 1-ml pipette, the sample was washed by adding 1 ml 1 × PBS solution and mixing well by vortexing. The sample was centrifuged at 18,000 g for 10 min, and then the supernatant was removed. After centrifuging at 18,000 g for 1 min, the additional liquid was removed using a 200-µl pipette, and the sample was dried at room temperature for 15 min. DNA from the mosquito leg was eluted by adding 80 µl of 20% Chelex solution and incubated for 10 min on a heating block shaker (Thomas Scientific, Swedesboro, NJ). After centrifuging at 18,000 g for 1 min, the supernatant (DNA) was transferred into a fresh collection tube and stored at − 20 °C until use.

### Molecular identification of *Anopheles* species

Multiplex PCR was used for the accurate identification of *Anopheles* species in the *Funestus* group (*An. minimus* subgroup and *An. funestus* subgroup) [29], the *Hyrcanus* group [30], the *Annularis* group [31], and the *Barbirostris* group [32]. PCR and DNA sequencings of the internal transcribed spacer region (ITS2) of the nuclear ribosomal DNA were also carried out for a subset of samples to confirm the species identified by multiplex PCR and for all samples not determined by the multiplex PCR method. Amplification of the ITS2 region was performed using the primer pair ITS2A (5′-TGT GAA CTG CAG GAC ACA T-3′) and ITS2B (5′-TAT GCT TAA ATT CAG GGG GT-3′) [33]. The detailed PCR conditions and DNA sequencing procedures have been described previously [34].

### Detection of *kdr* mutations

The *kdr* mutation of the voltage-gated sodium channel gene at codon L1014 residue was examined by PCR and DNA sequencing for all *Anopheles* species (*n* = 338) using the universal primer pair Ag-F (5'-GAC CAT GAT CTG CCA AGA TGG AAT-3') and An-kdr-R2 (5'-GAG GAT GAA CCG AAA TTG GAC-3') [35]. The PCR was conducted in a total reaction volume of 17 μl, including 2 μl of DNA template, 5 pmol of each primer, and 8.5 μl of DreamTaq Green PCR Master Mix (2X) (Thermo Fisher Scientific, Waltham, MA, USA). The thermocycling protocol consisted of an initial activation step of 3 min at 95 °C, followed by 35 amplification cycles of 30 s at 94 °C, 30 s at 55 °C, 45 s at 72 °C, and a final extension step of 6 min at 72 °C. Purification and DNA sequencing of PCR products were previously described [34].

### Data analysis

Mortality rates of adult female mosquitoes were calculated after the bioassays for each species group. Abbott’s formula was used to correct mortality rates if mortality in the control tube was between 5 and 20% [36]. Tests with > 20% mortality in the control were excluded from the analysis. Time to 50% knockdown (KDT50) and its confidence interval for each *Anopheles* species were determined by the log-probit method described by Finney [37] using the Excel spreadsheet calculator [38]. Analysis of variance (ANOVA) was performed to determine differences in mortality rates among species and between the two sampling methods.

The CodonCode Aligner 9.0.1 (CodonCode Corp., Centerville, MA) was used to check the sequence quality and trim low-quality bases. Bio-Edit software was used to align the sequences and to calculate pairwise sequence identity and similarity from multiple sequence alignments. A threshold limit of 98% sequence similarity for ITS2 was used to classify sequences into species [34]. The consensus sequences were compared to the NCBI nr/nt database (https://blast.ncbi.nlm.nih.gov/Blast.cgi). Phylogenetic analyses were performed using UPGMA with the Kimura 2-parameter model for ITS2 and *kdr* sequences. The tree nodes were evaluated by bootstrap analysis for 1000 replicates.

## Results

### Insecticide susceptibility bioassays

**Fig. 2:**
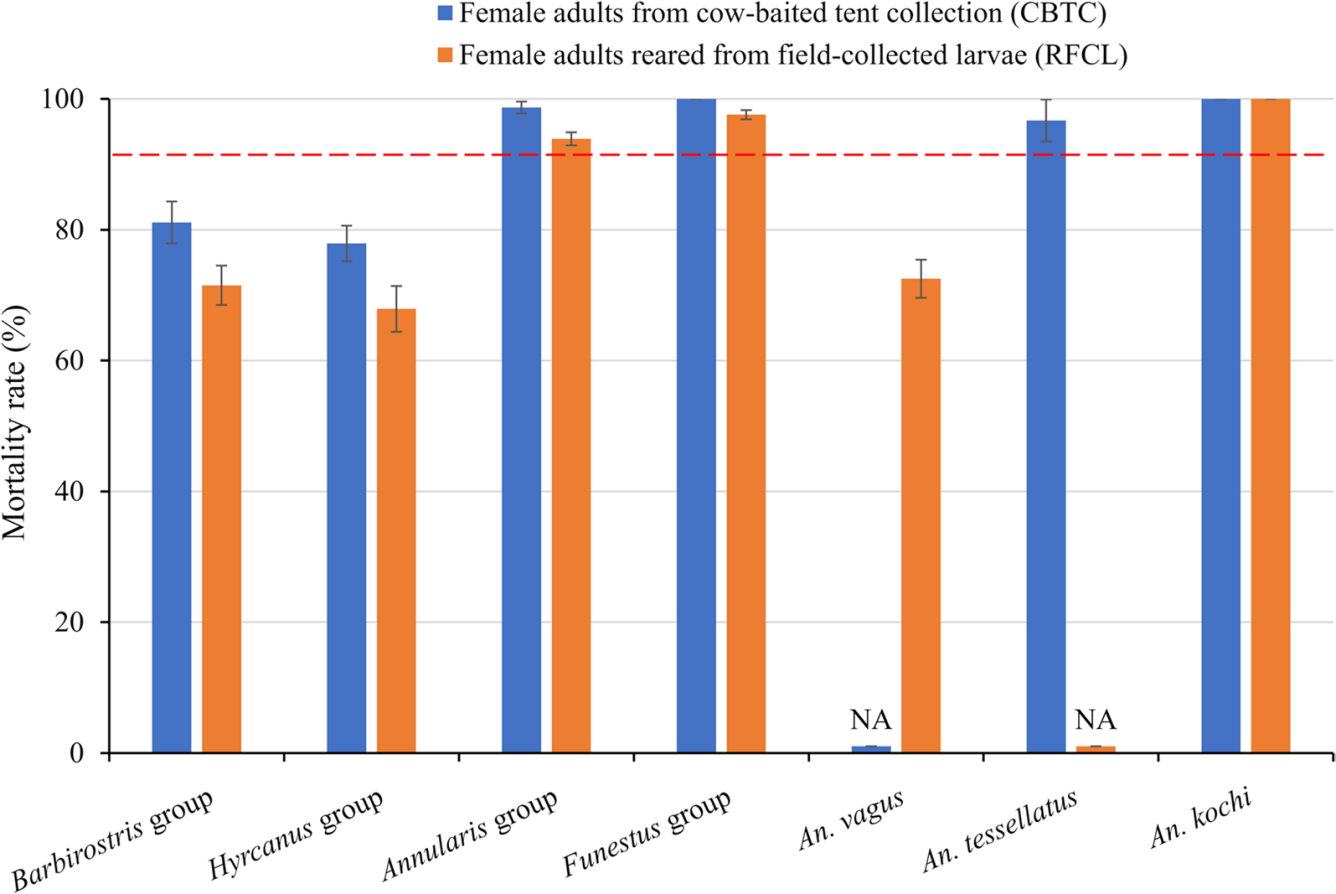
Mortality rate of *Anopheles* mosquitoes exposed to deltamethrin following the WHO susceptibility test procedure for insecticide monitoring in malaria vectors. The mortality rate was recorded after a 24-h recovery period. NA, not available. Red dashed line indicates cutoff for confirmed resistance (< 90%) (WHO 2018 update). *Anopheles sinensis* susceptible laboratory strain showed 100% mortality

A total of 868 adult females were collected from cow-baited tents and separated based on morphology and abundance (minimum sample size > 50) into six main species groups (*Barbirostris* group, *Hyrcanus* group, *Annularis* group, *Funestus* group, *An. tessellatus*, and *An. kochi*) and others. Over 5000 *Anopheles* mosquito larvae and pupae were collected from the three study villages. A total of 770 adult females were reared from field-collected larvae (RFCL) and classified into six different species groups (*Barbirostris* group, *Hyrcanus* group, *Annularis* group, *Funestus* group, *An. vagus*, and *An. kochi*) and others. Deltamethrin susceptibility bioassays were performed on adult females from both CBTC and RFCL based on their species group. The mortality of mosquitoes without exposure to insecticide was < 5% in all control tubes. The laboratory strain of *An. sinensis*, when exposed to the insecticide, showed average mortality of > 99%. Deltamethrin resistance was found in mosquitoes of the *Barbirostris* group and the *Hyrcanus* group from both CBTC (mortality rate 77.9–81.1%) and RFCL (mortality rate 67.9–71.5%) as well as in *An. vagus* from RFCL (mortality rate 72.5 ± 2.9%) (Table 1, Fig. 2). Possible resistance to deltamethrin was found in mosquitoes of the *Minimus* group and the *Annularis* group from RFCL and in *An. tessellatus* from CBTC, while *An. kochi* mosquitoes from both CBTC and RFCL were found susceptible to deltamethrin. A significant difference in mortality rate was found among species (ANOVA, *F*_(6,85)_ = 12.7, *P* < 0.001). Significantly lower mortality rates were found in adult females reared from field-collected larvae compared to those from CBTC in all species combined (ANOVA, *F*_(1,90)_ = 6.7, *P* < 0.01) and in four out of the seven species groups (ANOVA, *F*_(1,56)_ = 3.8, *P* < 0.05) (Table 1). The knockdown time ranged from 11.6 to 24.9 min in mosquitoes from CBTC and from 15.8 to 26.6 min in mosquitoes from RFCL (Table 1).

**Table 1:** Mortality rates and knockdown times of *Anopheles* mosquitoes exposed to deltamethrin in Myanmar

Species group	Female adults from cow-baited tent collection (CBTC)				Female adults reared from field-collected larvae (RFCL)				*P*-value
	*n*	KT50/min (95% CI)	MR (%) (mean ± SE)	Status	*n*	KT50/min (95% CI)	MR (%) (mean ± SE)	Status	
*Barbirostris*	170	24.9(22.4–27.6)	81.1 ± 3.2	R	166	26.6(24.3–29.2)	71.5 ± 3.0	R	0.0462
*Hyrcanus*	260	16.5(14.8–18.5)	77.9 ± 2.7	R	168	21.1(18.7–23.7)	67.9 ± 3.5	R	0.0241
*Annularis*	144	17.6(15.4–20.1)	98.7 ± 0.9	S	115	18.8(16.8–21.2)	93.9 ± 1.0	PR	0.0052
*Funestus*	110	11.6(10.4–13.0)	100	S	148	15.8(14.1–17.6)	97.6 ± 0.7	PR	0.0426
*Vagus*	0	na	na	na	102	20.6(18.4–23.1)	72.5 ± 2.9	R	na
*Tessellatus*	93	13.3(11.5–15.3)	96.7 ± 3.2	PR	0	na	na	na	na
*Kochi*	64	12.9(11.1–14.9)	100	S	56	19.3(17.5–21.5)	100	S	na

KT50, time to knockdown 50% mosquitoes; MR, mortality rate; CI, confidence interval; S, susceptible (mortality rate ≥ 98%); SE, standard error; PR, probably resistant (mortality rate 90–97%); R, resistant (mortality rate < 90%). Values for *P* < 0.05 indicate significant difference in mortality rates between the two sampling methods (CBTC and RFCL). An *An. sinensis* susceptible laboratory strain was used as a control for comparison

### Molecular identification of *Anopheles* species

**Fig. 3:**
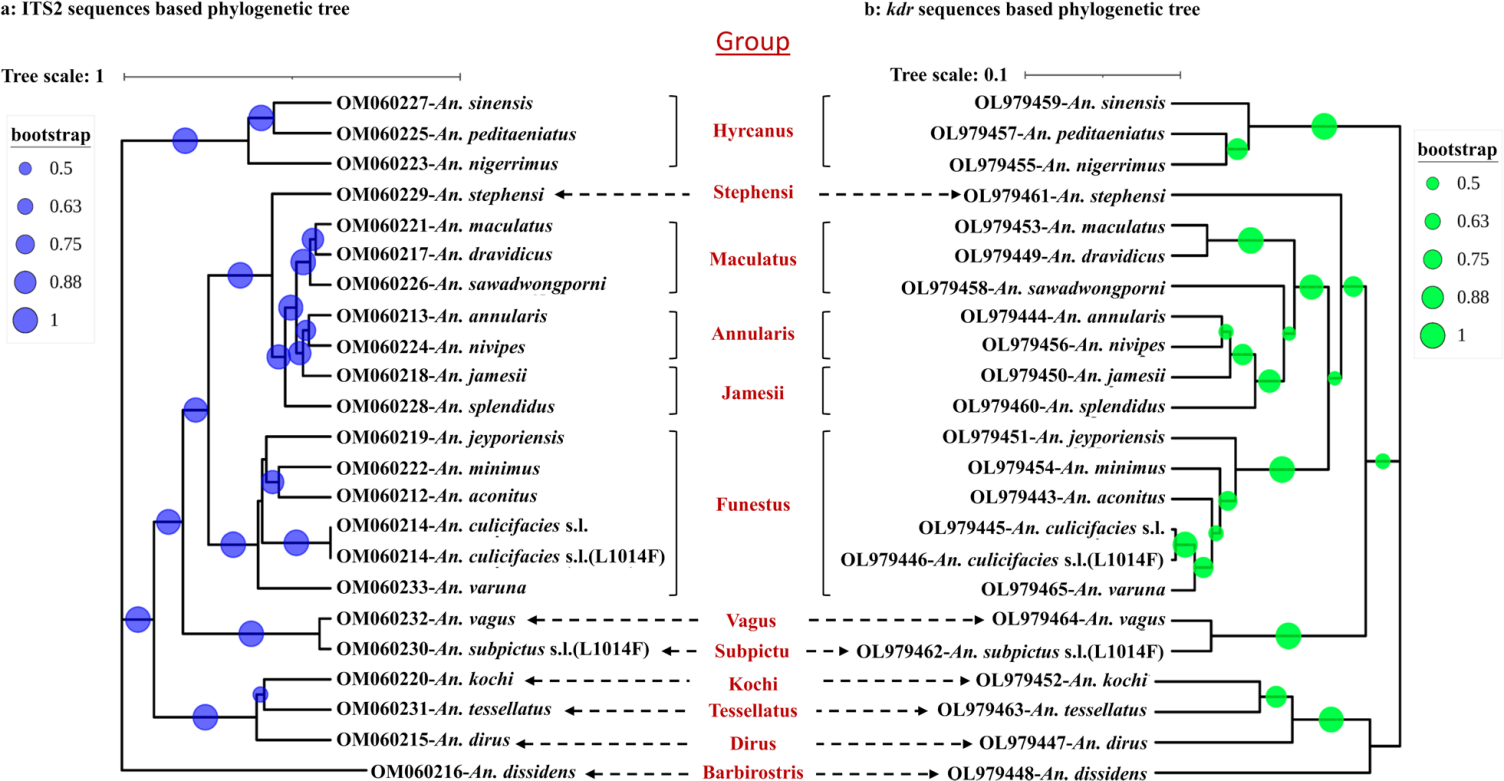
Phylogenetic tree inferred from UPGMA method based on ITS2 sequences (**a**) and *kdr* sequences (**b**). Bootstrap support values ≥ 50% are displayed by colored dots next to the branches. Tips of the tree are labeled as GenBank accession followed by *Anopheles* species identified in this study

The bioassayed mosquito samples from four species groups were genotyped by multiplex PCR and classified into seven species: *An. dissidens* (formerly *Anopheles barbirostris* species A1) (*n* = 336), *An. peditaeniatus* (*n* = 318), *An. aconitus* (*n* = 220), *An. nivipes* (*n* = 189), *An. sinensis* (*n* = 108), *An. annularis* (*n* = 70), and *An. minimus* (*n* = 28) (Table 2). PCR and DNA sequencing of the ITS2 region confirmed the identities of these species (*n* > 20 per species) and identified four additional species: *An. nigerrimus* (*n* = 2) from the *Hyrcanus* group and *An. culicifacies* (s.l.) (*n* = 10), *An. varuna* (*n* = 3), and *An. jeyporiensis* (*n* = 1) from the *Funestus* group. The ITS2 sequences of *An. culicifacies* (s.l.) showed > 99% similarity to multiple subspecies: *An. culicifacies* B, C, and E (GenBank AJ534247, AJ534643, and AJ534645). Thus, the accurate subspecies of this complex could not be determined by the ITS2 sequences. All specimens from each of the three species groups *An. kochi*, *An. tessellatus*, and *An. vagus* were confirmed by ITS2 sequencing. In addition, 42 specimens of the minor species not used for bioassays were also sequenced and classified into eight *Anopheles* species from five groups (*Jamesii, Maculatus, Dirus, Subpictus, and Stephensi*) compared to the ITS2 sequences retrieved from GenBank (Table 2). The ITS2 sequences of *An. subpictus* (s.l.) showed > 99% similarity to *An. subpictus* A cluster (GenBank KC191825), which includes multiple biological forms: *An. subpictus* A, C, and D. Thus, the accurate biological forms of this complex could not be determined by the ITS2 sequences. Phylogenetic analysis indicated that species from the same species group were generally clustered together with large bootstrap values, such as for the *Hyrcanus, Maculatus*, and *Funestus* groups (Fig. 3a). The *Barbirostris* group included only one species, *An. dissidens*, with the longest ITS2 sequence length (1.8 k), which fell into a different cluster in the phylogenetic tree.

**Table 2:** Molecular identification of species composition and abundance in northern-central Myanmar

Species group	Species	CBTC		FCL		Total	%
		R	S	R	S		
*Barbirostris*	*An. dissidens*	32	138	46	120	336	20.5
*Hyrcanus*	*An. peditaeniatus*	57	157	24	80	318	19.4
	*An. sinensis*	0	44	0	64	108	6.6
	*An. nigerrimus*	0	2	0	0	2	0.1
*Annularis*	*An. annularis*	2	48	0	20	70	4.3
	*An. nivipes*	0	94	7	88	189	11.5
*Funestus*	*An. culicifacies* (s.l.)	0	2	4	4	10	0.6
	*An. aconitus*	0	102	0	114	216	13.2
	*An. minimus*	0	4	0	24	28	1.7
	*An. varuna*	0	1	0	2	3	0.2
	*An. jeyporiensis*	0	1	0	0	1	0.1
*Vagus*	*An. vagus*	0	0	28	74	102	6.2
*Tessellatus*	*An. tessellatus*	3	90	0	0	93	5.7
*Kochi*	*An. kochi*	0	64	0	56	120	7.3
*Jamesii*	*An. jamesii*	3		5		8	0.5
	*An. splendidus*	5		6		11	0.7
*Maculatus*	*An. sawadwongporni*	4		1		5	0.3
	*An. maculatus*	3		1		4	0.2
	*An. dravidicus*	1		0		1	0.1
*Dirus*	*An. dirus*	7		2		9	0.5
*Subpictus*	*An. subpictus* (s.l.)	2		0		2	0.1
*Stephensi*	*An. stephensi*	2		0		2	0.1
	Total	868		770		1638	100.0

CBTC, female adults from cow-baited tent collection; RFCL, female adults reared from field-collected larvae; R, resistant; S, susceptible

### *Kdr* mutations in the voltage-gated sodium channel gene

In total, 338 specimens from 22 *Anopheles* species were successfully sequenced for the *kdr* genes covering codon L1014. The L1014F *kdr* mutation was detected in the homozygous state in two *An. subpictus* (s.l.) specimens and in the heterozygous state in one *An. culicifacies* (s.l.) specimen. All other species were wild type at the *kdr* L1014 locus (Additional file 1: Fig. S1). Phylogenetic analysis of the 22 *Anopheles* species indicated that the *kdr* sequences generally clustered together within the same species group with large bootstrap values and showed a similar pattern with that inferred from the UPGMA method based on the ITS2 sequences (Fig. 3b).

## Discussion

Understanding vector community structure, species distribution, vector abundance, and insecticide resistance is essential for developing cost-effective strategies to control malaria vectors. This study provides updated information about *Anopheles* species composition and abundance surveyed from adult and larva collections of field mosquito populations and, for the first time to our knowledge, reports the insecticide resistance of seven species or species complexes in northern-central Myanmar. The *kdr* primer set was confirmed to work for all the *Anopheles* species identified in this study.

Out of 22 *Anopheles* species identified molecularly, four (*An. dissidens, An. peditaeniatus, An. aconitus*, and *An. nivipes*) were the most common (accounting for ~ 65%), while the rest were below 10% each. Previous surveillance of malaria vectors conducted in the nearby township (Indaw, Saging Division) identified 19 *Anopheles* species, with *An. philippinensis, An. fluviatilis* (s.l.), *An. jamesii*, and *An. vagus* as the four most common species based on morphological identification [21]. However, this study did not find four previously reported species (*An. philippinensis, An. fluviatilis, An. karwari*, and *An. majidi*), possibly because of the short duration for sample collection or different sampling methods used. In contrast, this study identified seven previously unreported species (*An. dirus, An. peditaeniatus, An. nigerrimus, An. nivipes, An. sawadwongporni, An. dravidicus*, and *An. jeyporiensis*), which were further confirmed by molecular identification. The changes in species composition and abundance may reflect local environmental changes causing species to shift their habitat range, biting and resting behaviors, and host choice as they track their ecological niches [39].

The widespread use of insecticide-treated nets (ITNs) and IRS has been shown to alter the species composition, abundance, and behavior of vector populations [40, 41]. In Thailand, IRS resulted in a higher proportional decrease of *An. dirus* (s.l.) compared to *An. minimus* (s.l.) [42], whereas ITN use in China caused a more substantial decrease of the endophilic and anthropophilic *An. lesteri* (synonym: *An. anthropophagus*) [43] and *An. minimus* (s.l.) [44] than of the exophagic and zoophilic *An. sinensis*. In Nepal, residual spraying has proved to be effective against indoor resting species, e.g. *An. annularis, An. culicifacies* (s.l.), *An. splendidus* and *An. vagus*, and some outdoor resting species, e.g. *An. fluviatilis* (s.l.) and *An. maculatus* (s.l.) [45]. Shifts of malaria vectors in biting behavior such as shifts to outdoor or early biting and shifts to zoophily or exophily have been observed in *An. dirus* (s.l.) and *An. minimus* (s.l.) of Thailand [42, 46, 47]. The level of behavioral heterogeneity of *Anopheles* species and populations is much higher in Southeast Asia than on the other continents according to ecological situations [48, 49]. These heterogeneities of species and behavioral shifting may result in differences in epidemiological importance and will have a great potential to increase malaria transmission in regions with a high coverage of ITNs and IRS [40, 41, 50]. Thus, there is an urgent need for additional control measures tackling malaria transmission by these vector populations.

Previous parasitological studies identified by morphology only one primary vector (*An. minimus*) and six secondary vectors (*An. aconitus, An. annularis, An. culicifacies* (s.l.), *An. sinensis, An. maculatus*, and *An. philippinensis*) in Indaw township [21]. In this study, *An. dirus* (a primary vector in Southeast Asia) was also found at low density. Whereas the secondary vector *An. philippinensis* was not detected, the closely related, morphologically similar species *An. nivipes* of the *Annularis* group was collected in Banmauk township. These results may reflect the effects of seasonality, sampling methods, and sampling duration in species composition and richness. Among the four common species identified in this study, *An. dissidens* (formerly *An. barbirostris* species A1), currently known from Thailand, China, and Myanmar [51], has been reported as a potential vector of *P. vivax* in Thailand [52, 53]. *Anopheles peditaeniatus* of the *Hyrcanus* group has been reported as a secondary vector of Japanese encephalitis virus in China and India [54–56] and a potential vector of *Brugia malayi* filarial worms [57]. *Anopheles peditaeniatus* has also been reported to be positive for *P. falciparum* infection by ELISA in Sri Lanka [58], Thailand [57, 59], and Indonesia [60]. The common species *An. nivipes* in Banmauk is a recognized malaria vector in Bangladesh, Thailand, and India [61]. The rare species *An. nigerrimus* of the *Hyrcanus* group in Banmauk was also recognized as a suspected vector of malaria in Bangladesh [62].

Out of the seven *Anopheles* groups tested in this study, three (*Barbirostris, Hyrcanus*, and *Vagus*) showed resistance to the pyrethroid insecticide deltamethrin, suggesting the presence of insecticide resistance in malaria vectors from northern Myanmar. *Anopheles dissidens* is the only common species detected in the *Barbirostris* group from Banmauk, whereas three species in the *Hyrcanus* group were found, with *An. peditaeniatus* being the predominant (~ 3/4), followed by *An. sinensis* (~ 1/4) and *An. nigerrimus* (< 1%). Resistance to deltamethrin has been previously reported in the *Barbirostris* group, the *Hyrcanus* group, and *An. vagus* from the Thai-Myanmar border on the Myanmar side [11] and in the *Hyrcanus* group from China [12–15, 50] and northeast Thailand [9] as well as in *An. vagus* from Bangladesh [63]. In the *Hyrcanus* group, all the resistant specimens were *An. peditaeniatus*, consistent with those findings from northeast Thailand [10]. However, the *Annularis* group, the *Funestus* group, *An. tessellatus*, and *An. kochi* were found to be susceptible (in the adult collection) or possibly resistant (in the larvae collection), similar to those detected in the Thai-Myanmar border area [11]. In the Sagaing Division of Myanmar, deltamethrin resistance in the *Hyrcanus* group has also been confirmed by the WHO, while other *Anopheles* species are susceptible or remain to be tested [64].

Previous findings showed that mosquito sampling and preparation methods significantly affected the mortality rates in the standard WHO tube resistance bioassay [65–67]. In Africa, Oliver, et al. (2014) reported that pyrethroid resistance levels in laboratory-reared non-blood-fed *An. arabiensis* females steadily decreased with age, whereas blood-fed females could significantly increase or maintain resistance compared to non-blood-fed females at the same age. Similar results were also found in the adult females reared from field-collected larvae of *An. gambiae* in western Kenya [67]. This study found that field-collected mixed-age blood-fed adult females of all four *Anopheles* species groups had significantly increased mortality rates against deltamethrin compared to non-blood-fed adult females reared from the larvae collection, consistent with a previous report of *An. sinensis* populations from central China [65]. Therefore, to minimize the effect of confounding factors such as mosquito age and blood-feeding status, the use of non-blood-fed adult 3–5 day-old female *Anopheles* is recommended to assess the susceptibility of field malaria vector populations to insecticides [5].

The insecticide resistance mechanism is still largely unknown in most *Anopheles* vector species from the GMS. Previous findings showed that both target site mutations (*kdr*) and metabolic detoxification play important roles in the deltamethrin resistance of *An. sinensis* field populations from China [12–15]. In *An. peditaeniatus*, a high allele frequency of L1014F *kdr* mutation (97.6%) was reported in Vietnam [68], whereas the L1014S *kdr* mutation was found in Vietnam, Thailand, and the Thai-Myanmar border at low frequencies [10, 11, 68]. The L1014S *kdr* mutation was also found in *An. vagus, An. sinensis*, and *An. paraliae* at low frequencies in Vietnam, but not in Thailand or Myanmar [10, 11, 68]. Among the 22 *Anopheles* species tested in this study, the *kdr* mutation (L1014F) was only detected in *An. subpictus* (s.l.) and at a low allele frequency in *An. culicifacies* (s.l.). The lack or scarcity of target-site resistance mutations suggested the involvement of other resistance mechanisms. The other resistane mechanisms include metabolic resistance by increased activities of detoxification enzymes (e.g. cytochrome P450 monooxygenases, glutathione S-transferase, and non-specific esterases), penetration resistance through thickening or remodeling of the cuticle to prevent the absorption or penetration of insecticide, and behavioral resistance by a change from their normal activity to avoid contact with or ingestion of a lethal dose of an insecticide [69]. Of the six families of P450s (CYP4, 6, 9, 12, 18, and 28), the CYP6 family has been well documented as being involved in insecticide resistance in the malaria vector *An. gambiae* [70–72]. Overexpression of cytochrome P450 isoforms (CYP6AA2, CYP6AA3, and CYP6P7) was also observed in laboratory-selected pyrethroid-resistant *An. minimus* mosquitoes in Thailand [73, 74].

This study has some limitations. First, we only conducted *Anopheles* resistance monitoring in a short period of time in three villages, considering the difficulty of accessing the study sites in northern central Myanmar. Second, identifying malaria vectors at the species level based on morphological traits remains a challenge due to the labor-intensiveness and difficulty separating species from groups or species complexes that are morphologically indistinguishable. Third, we could not collect enough mosquitoes of less abundant species (or species complex) of *Anopheles* for bioassay. Thus, only seven predominant species were assessed for susceptibility to insecticide in the study. Future studies should consider including testing different insecticides, collecting mosquitoes using multiple methods across different seasons, and covering a larger region to obtain a clear picture of the insecticide resistance situation in Myanmar. Molecular identification of *Anopheles* species should also be strengthened so that more species can be identified by the regular PCR method.

Bioassay and molecular monitoring of insecticide resistance status in local malaria vectors are crucial for resistance management and vector control. This study reported the presence of deltamethrin resistance in three *Anopheles* species complexes [*An. barbirostris* (s.l.), *An. hyrcanus* (s.l.), and *An. vagus*] tested in northern Myanmar, suggesting that these species complexes are probably subjected to the high selective pressure of insecticides used in the local ecological environment [75]. In Myanmar, pyrethroid insecticides have been intensively used for malaria control in the interior and along the periphery of human habitation areas since 1992 [76]. The use of pyrethroid insecticides in vector control and agricultural practices has potentially exerted selection pressure for resistance in mosquito populations, which may explain the deltamethrin resistance detected in the *Anopheles* complexes in this study [75]. Several strategies have been recommended to manage the resistance evolution in vector populations against chemical insecticides, such as minimizing insecticide use, avoiding persistent chemicals, using insecticide mixture or insecticide mosaic, and rotating insecticides with different chemical classes or different control methods [3].

## Conclusions

This study updated *Anopheles* species composition, vector abundance, and insecticide resistance status in northern Myanmar. The presence of confirmed resistance to pyrethroid insecticide and the absence of knockdown mutation (*kdr*) in *An. dissidens, An. peditaeniatus*, and *An. vagus* have important implications in pyrethroid-based vector control and resistance management. To identify alternatives to pyrethroids for vector control, further studies are needed to document the non-target-site resistance mechanisms (e.g. rapid metabolic detoxification) and the susceptibility status of malaria vectors to other classes of insecticides (e.g. carbamates, organophosphates, and insect growth regulators).

## Supplementary Information

**Additional file 1: Figure S1.** Partial sequences showing the *kdr* L1014 allele (highlighted) in the 22 *Anopheles* species from northern-central Myanmar. Numbers in parentheses indicate the sample size.

## Data Availability

The datasets used and/or analyzed during the current study are available from the corresponding author upon reasonable request. The ITS2 sequences obtained in the study are available in GenBank with accession numbers OM060212–OM060233. The *kdr* sequences obtained in the study are available in GenBank with accession numbers OL979443–OL979465.

## Abbreviations

GMS: Greater Mekong Subregion
WHO: World Health Organization
COVID: Coronavirus Disease
CBTC: Female adults from cow-baited tent collection
RFCL: Female adults reared from field-collected larvae
*kdr*: Knockdown resistance
KT50: Time to knockdown 50% mosquitoes
MR: Mortality rate
CI: Confidence interval
S: Susceptible
PR: Probably resistant
R: Resistant
ITS2: Ribosomal internal transcribed spacer 2
PCR: Polymerase chain reaction
DDT: Dichlorodiphenyltrichloroethane
ANOVA: Analysis of variance
UPGMA: Unweighted pair group method with arithmetic mean
