# Supplementary material for: Community structure and insecticide resistance of malaria vectors in northern-central Myanmar
Source: Parasit Vectors. 2022 May 3;15:155. doi: 10.1186/s13071-022-05262-2 (PMC9062858; doi:10.1186/s13071-022-05262-2)
Supplement: Supplementary file 1 — Additional file 1: Figure S1. Partial sequences showing the kdr L1014 allele (highlighted) in the 22 Anopheles species from northern-central Myanmar. Numbers in parentheses indicate the sample size. [file 13071_2022_5262_MOESM1_ESM.pdf]

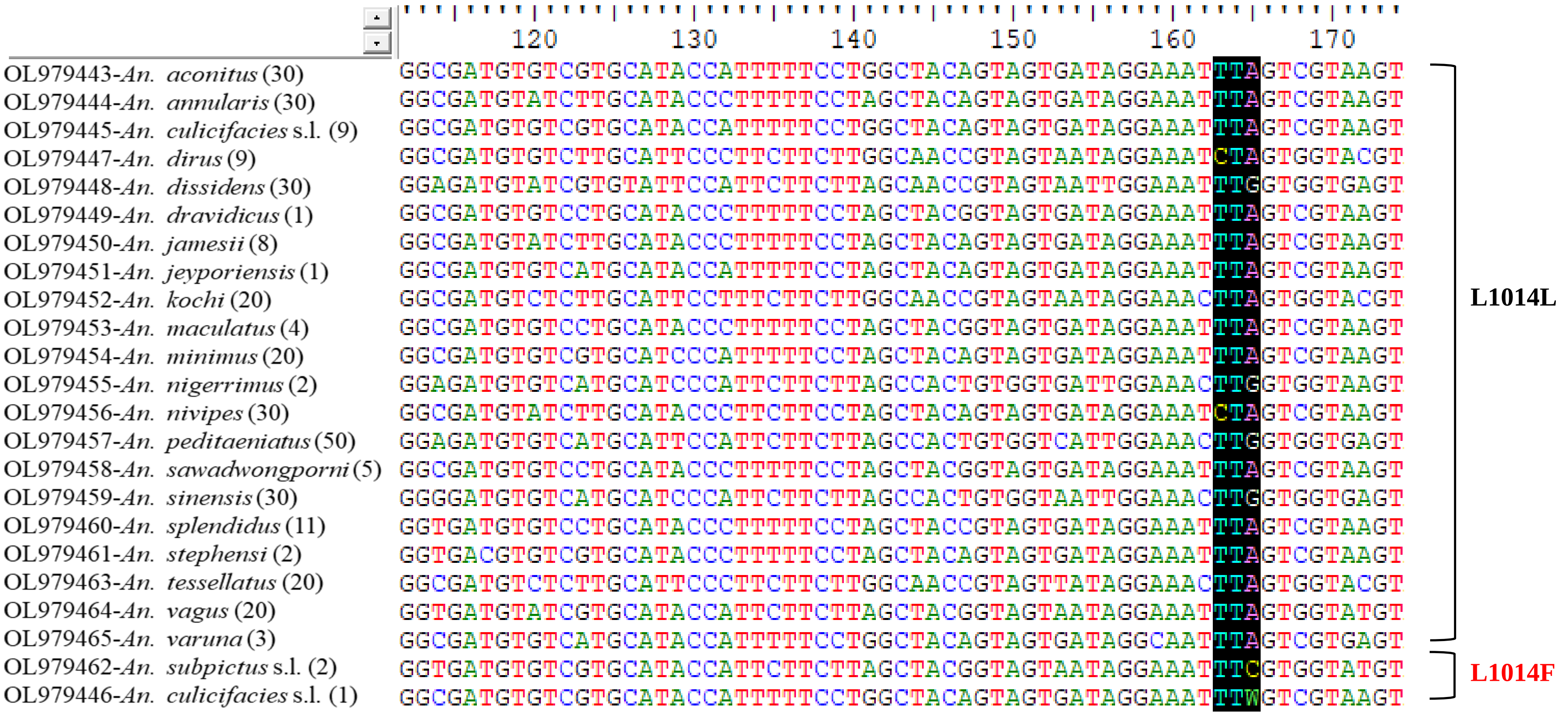

Figure S1 Partial sequences showing the *kdr* L1014 allele (highlighted) in the 22 *Anopheles* species identified in the northern-central Myanmar. Numbers in parentheses indicate the sample size. GenBank accession: OL979443-OL979465.
